# Evaluation of treatment outcomes for AML patients ineligible for intensive chemotherapy before and after the implementation of VEN/AZA – a retrospective cohort study

**DOI:** 10.1515/med-2026-1499

**Published:** 2026-08-18

**Authors:** Norea Gruber, Josef Singer

**Affiliations:** Karl Landsteiner University of Health Sciences, Krems an der Donau, Austria; Department of Internal Medicine II, University Hospital Krems, Karl Landsteiner University of Health Sciences, Krems an der Donau, Austria

**Keywords:** acute myeloid leukemia, azacitidine, venetoclax, real-world data, survival analysis

## Abstract

**Objectives:**

Acute myeloid leukemia (AML) predominantly affects older patients, many of whom are ineligible for intensive chemotherapy. Although the VIALE-A trial demonstrated improved survival with azacitidine plus venetoclax, its strict eligibility criteria may limit generalizability to routine clinical practice. We therefore evaluated real-world outcomes at our institution.

**Methods:**

We conducted a retrospective single-centre cohort study of adult AML patients ineligible for intensive chemotherapy. Patients received azacitidine monotherapy (2014–2018) or azacitidine plus venetoclax (2019–2023). Overall survival (OS) and progression-free survival (PFS) were analyzed using Kaplan-Meier estimates and log-rank tests. VIALE-A eligibility was assessed retrospectively.

**Results:**

21 patients met the inclusion criteria: 8 received azacitidine alone and 13 azacitidine plus venetoclax. Median age was 79 years. Patients receiving azacitidine plus venetoclax completed more treatment cycles (median 7 vs. 4). Median OS was 12.6 vs. 9.1 months with azacitidine alone (p=0.416). Median PFS was 10.0 vs. 8.1 months, respectively (p=0.707). Overall, only 66 % of patients fulfilled the VIALE-A eligibility criteria.

**Conclusions:**

Azacitidine plus venetoclax was associated with numerically longer OS and PFS, although differences were not statistically significant. Only two-thirds of patients would have met VIALE-A eligibility criteria, highlighting the challenges of translating trial outcomes into routine practice. Larger multicentre studies are warranted.

## Introduction

Acute myeloid leukemia (AML) is a heterogeneous disease of myeloid stem cell precursors, primarily affecting elderly individuals, with a median age at diagnosis of 68 years. Standard treatment consists of intensive induction chemotherapy followed by consolidation chemotherapy and/or allogeneic stem cell transplantation [1]. However, older patients often respond poorly due to adverse genomic features, higher treatment resistance, and frequent ineligibility for intensive regimens caused by age, comorbidities, or organ dysfunction [2], [3], [4]. Consequently, they typically receive lower-intensity therapies such as hypomethylating agents (azacitidine, decitabine) or low-dose cytarabine [5], 6]. Historically, remission rates in this group were ≤30 %, with a median survival below 12 months [1].

The VIALE-A trial demonstrated that combining azacitidine with venetoclax improved progression-free and overall survival in patients ineligible for intensive therapy [7]. However, its strict inclusion and exclusion criteria limit generalizability, and real-world patients may experience higher toxicity rates [8].

This study aimed to evaluate the effectiveness of azacitidine plus venetoclax compared with the former standard of care, azacitidine monotherapy in a real-world setting.

### Two key research questions were thus investigated

– What are the differences in progression-free survival (PFS) and overall survival (OS) before and after the introduction of azacitidine plus venetoclax for AML patients ineligible for intensive chemotherapy at the University Hospital Krems?
– What proportion of real-world patients treated at the University Hospital Krems would have met the strict inclusion and exclusion criteria of the VIALE-A trial?

## Materials and methods

### Study design and population

We conducted a retrospective cohort study analyzing patients’ data from the data management system of the University Hospital Krems “Medical Process Administrator” (MPA) and the Oncology Information System (OIS) of Lower Austria, a database, that undergoes active, systematic quality control, for which previous publications have demonstrated its suitability for robust, peer-reviewed analyses [9], 10].

All patients diagnosed with AML and ineligible for standard intensive chemotherapy, who were treated with azacitidine monotherapy starting between 01.01.2014 and 31.12.2018 or with the combination therapy azacitidine and venetoclax starting between 01.01.2019 and 31.12.2023 at the University Hospital Krems, were screened for the following criteria:

### Inclusion criteria

– Patients diagnosed with AML by WHO criteria and ineligible for treatment with an intensive chemotherapy induction regimen due to age or co-morbidities

### Exclusion criteria

– Patients under the age of 18 years
– Patients without AML
– Patients with another specific first line treatment or another cancer
– Patients with MDS previously treated with azacitidine monotherapy

The observation period extended until 30.06.2024.

### Data collection

Patient data were obtained from the clinical data management system “Medical Process Administrator” (MPA) and the Lower Austrian registry “Oncology Information System” (OIS). Data were pseudonymized prior to analysis.

### Statistical analysis

Descriptive analyses included age, sex, treatment, cycle number, and VIALE-A eligibility. Outcomes were overall survival (OS) and progression-free survival (PFS). Overall survival was defined as the time from treatment initiation to death from any cause. Progression-free survival was defined as the time from treatment initiation to disease progression, relapse after response, or death from any cause, according to ELN response criteria. Groups were compared using Kaplan–Meier analysis and log-rank tests. Analyses were performed with SPSS (Version 29.0.2.0). Patients with incomplete therapy or response data were excluded; missing descriptive data were tolerated.

### VIALE-A eligibility

Eligibility for the pivotal VIALE-A trial was assessed according to the inclusion and exclusion criteria reported in the original publication [7].

### Research ethics

The study was approved by the Institutional Review Board and the Ethics Committee of the Karl Landsteiner University of Health Sciences (EK No. 1053/2024) and performed in accordance with the Declaration of Helsinki (as revised in 2013).

### Informed consent

Informed consent was waived due to the retrospective design.

## Results

### Patient characteristics

A total of 21 patients met the inclusion criteria, of whom 8 received azacitidine monotherapy and 13 were treated with azacitidine plus venetoclax. Baseline characteristics are shown in Table 1. Sex distribution was similar across both groups, with a slight predominance of male patients (62.5 % in the monotherapy group and 69.2 % in the combination group). The median age at diagnosis was comparable between groups (78.5 years [range 55–90] vs. 79 years [range 65–84]). Patients in the combination group received a higher median number of treatment cycles (7 vs. 4). With respect to VIALE-A eligibility, 62.5 % of patients in the monotherapy group and 69.2 % in the combination group would have met the inclusion criteria of the trial.

**Table 1:** Patient characteristics.

Parameter	Azacitidine monotherapy	Azacitidine + venetoclax therapy
Male n (%)	5 (62.5)	9 (69.2)
Female n (%)	3 (37.5)	4 (30.8)
Age in years at therapy start median (min – max)	78.5 (55–90)	79 (65–84)
ECOG performance status at therapy start median (min – max)	1 (0–4)	1 (0–2)
White blood cell count at therapy start Median G/L (min – max)	2.06 (1.46–71.70)	3.91 (1.21–17.99)
Blast percentage in peripheral blood at therapy start Median % (min – max)	4.55 (0–26)	0.3 (0–76)
Number of treatment cycles median (min – max)	4 (1–21)	7 (1–20)
VIALE-A eligibility n (%)	5 (62.5)	9 (69.2)

### Overall survival

**Figure 1:**
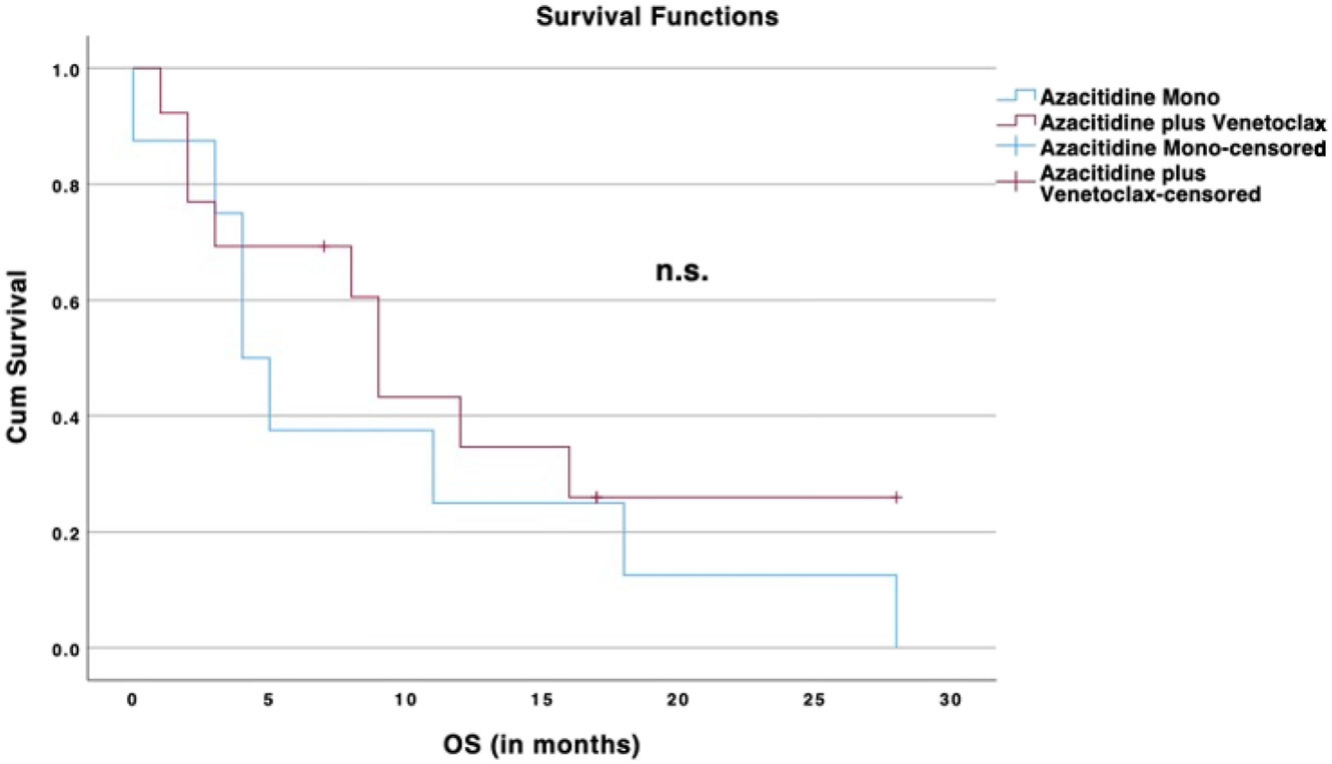
Overall survival (OS) of treatment cohorts. The median overall survival was 9.1 months for the azacitidine monotherapy group and 12.6 months for the combination of azacitidine and venetoclax group (not significant (n.s.), p=0.416).

Median overall survival (OS) was 9.1 months in the azacitidine monotherapy group compared to 12.6 months in the azacitidine plus venetoclax group (Figure 1). Although a numerical improvement was observed, the difference was not statistically significant (log-rank p=0.416). The Kaplan-Meier curves demonstrated an early separation favoring the combination regimen, but this benefit was not maintained to a statistically significant extent over time.

### Progression-free survival

**Figure 2:**
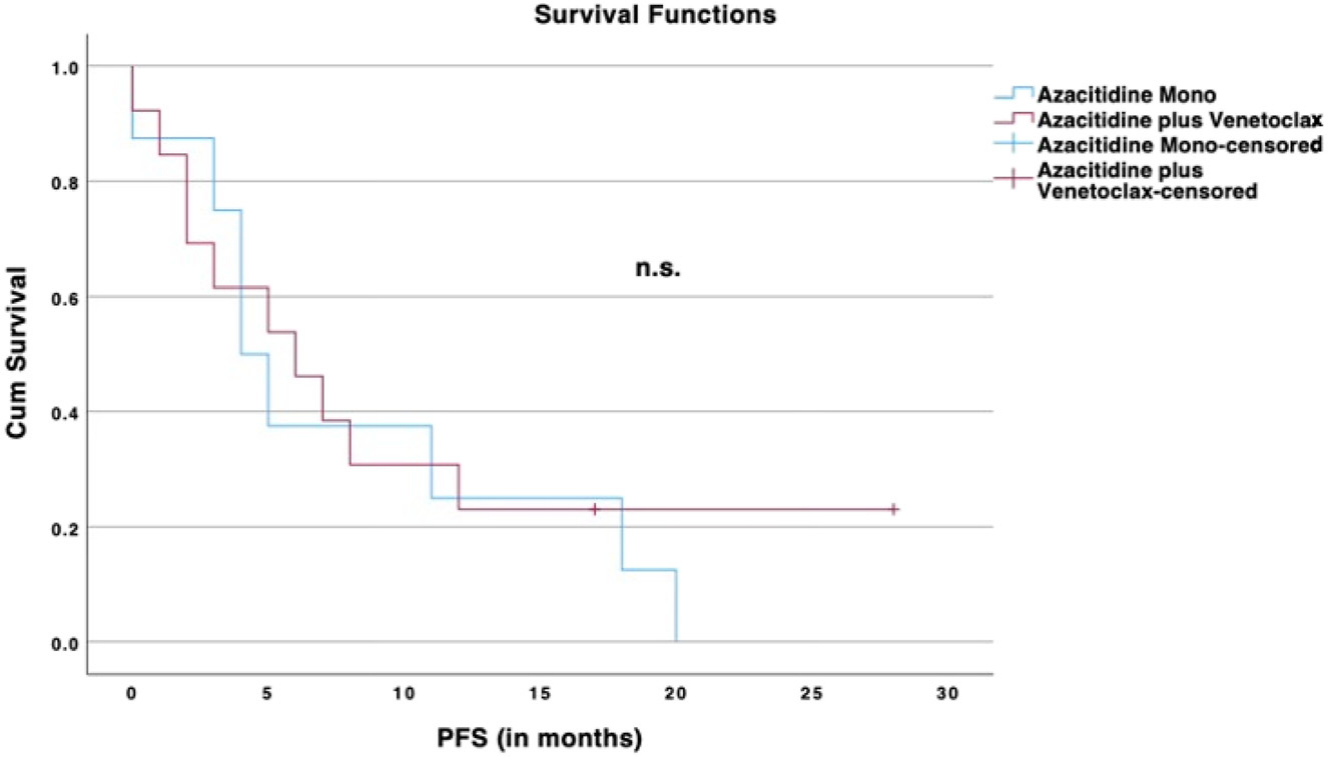
Progression-free survival (PFS) of treatment cohorts. The median progression-free survival was 8.1 months in the azacitidine monotherapy group and 10.0 months in the group receiving both azacitidine and venetoclax (not significant (n.s.), p=0.707).

Median progression-free survival (PFS) was 8.1 months with azacitidine alone vs. 10.0 months with the combination regimen (Figure 2). Similar to OS, the difference was not statistically significant (log-rank p=0.707).

### VIALE-A eligibility

When applying VIALE-A eligibility criteria to the study population, only two-thirds of patients in routine practice would have been eligible for enrollment in the trial. Specifically, 9 of 13 patients (69.2 %) in the combination group and 5 of 8 patients (62.5 %) in the monotherapy group met the trial’s strict inclusion and exclusion criteria.

The most common reasons for ineligibility were poor ECOG performance status and severe cardiac comorbidities.

## Discussion

In this retrospective cohort, the combination of azacitidine and venetoclax achieved numerically higher survival outcomes compared to azacitidine monotherapy. Median OS improved from 9.1 to 12.6 months, and median PFS from 8.1 to 10.0 months. However, neither difference reached statistical significance (p=0.416 for OS; p=0.707 for PFS). These findings show numerically longer survival with combination therapy, although no statistically significant difference was demonstrated in this small real-world cohort.

### Comparison with VIALE-A

The VIALE-A trial demonstrated statistically significant improvements in both OS and PFS for azacitidine plus venetoclax compared to azacitidine alone [7] and is thus regarded as the current standard of care for patients with newly diagnosed AML who are ineligible for intensive chemotherapy [11]. In contrast, our real-world cohort did not reproduce this level of significance. A possible explanation is patient selection: VIALE-A applied strict inclusion and exclusion criteria, excluding individuals with poor performance status or severe comorbidities. Our study reflects a more heterogeneous, frailer patient population. Table 2 and Table 3 summarize survival outcomes in our cohort compared to VIALE-A. This comparison is descriptive only and should be interpreted cautiously because of differences in study design, sample size, eligibility criteria, and patient characteristics.

**Table 2:** Comparison of OS.

Study	Median OS in azacitidine monotherapy	Median OS in azacitidine + venetoclax treatment
VIALE-A trial	9.6 months	14.7 months
Own study results	9.1 months	12.6 months

**Table 3:** Comparison of PFS.

Study	Median PFS in azacitidine monotherapy	Median PFS in azacitidine + venetoclax treatment
VIALE-A trial	7.0 months	9.8 months
Own study results	8.1 months	10.0 months

A recent systematic review of 73 real-world studies (2025) reported a pooled median OS of 9.8 months for azacitidine plus venetoclax – substantially lower than VIALE-A (14.7 months), but comparable to our cohort (12.6 months) [7], 8]. This highlights the well-known gap between clinical trial outcomes and real-world effectiveness.

Long-term follow-up of VIALE-A confirmed durable benefit with combination therapy (median OS 14.7 months; 24-month OS rate 37.5 %) but also underscored that neither treatment is curative without allogeneic stem cell transplantation, which only a small minority of patients could undergo [12]. Interestingly, a 2025 study explored reduced venetoclax exposure (“7 + 7 regimen”) in patients with hematological toxicity. Shortening treatment to 7 days per cycle yielded a median OS of 11.2 months, comparable to standard dosing (10.3 months), while reducing early mortality and transfusion needs [13]. Such adaptations may enhance applicability in real-world settings.

### VIALE-A eligibility in real-world patients

Only 69.2 % of patients treated at the University Hospital Krems with azacitidine plus venetoclax would have met VIALE-A eligibility, and 62.5 % in the monotherapy group. This underscores the selectivity of trial populations compared to real-world practice, and why translating trial results into real-world outcomes remains challenging.

### Limitations

The retrospective design carries inherent risks of information bias, as medical records may be incomplete or inconsistently documented. In this study, data on ELN risk classification, comorbidity scores, AML subtype, and comprehensive cytogenetic/molecular characteristics were not consistently available because of the retrospective nature of the study and the long inclusion period.

Furthermore, the single-center design and small sample size limited statistical power. Given the limited sample size (n=21) and the risk of model overfitting, we did not perform multivariable Cox regression analyses.

Data on remission rates, treatment-related toxicities and dose modifications were also not consistently available in our retrospective dataset and therefore could not be analyzed reliably.

Finally, because treatment cohorts were defined by different calendar periods, changes in supportive care, diagnostic approaches, and clinical management may have influenced outcomes independently of treatment regimen.

### Outlook

Further, larger studies are needed to optimize the administration of azacitidine and venetoclax in real-world practice, including dose modifications and patient selection strategies. Particular attention should be given to integrating genetic risk stratification, as novel combination therapies have been approved or under investigation. For example, patients with IDH1-mutated AML benefit most from ivosidenib plus azacitidine, achieving a median OS of 24.0 vs. 7.9 months in controls [14].

The new European LeukemiaNet (ELN) classification refines prognostic risk categories (favorable, intermediate, adverse) and may help identify subgroups best suited for lower-intensity regimens [15].

## Conclusions

At the University Hospital Krems, the combination of azacitidine and venetoclax was associated with numerically longer median OS and PFS compared with azacitidine monotherapy, but the differences were not statistically significant, which may in part be attributable to the small cohort size and limited statistical power of this single center analysis. Compared with VIALE-A, treatment outcomes in real-world practice may be attenuated, potentially reflecting differences in patient frailty and trial eligibility.

Multicenter studies with larger cohorts are warranted to clarify the true effectiveness of azacitidine plus venetoclax in unselected AML populations. Personalized treatment strategies based on genetic mutations and optimized dosing regimens will be essential to further improve outcomes.
